# A uniQue wAy of Transcatheter mAss extraction: The QATAR Procedure

**DOI:** 10.1016/j.jaccas.2025.103792

**Published:** 2025-07-03

**Authors:** Mhd Baraa Habib, Shabib AlAasmy, Cheikh Ahmed Aboulmaaly, Hakam Alzaeem, Nazar Mohammed, Mohammed Al-Hijji, Mohammed Qintar

**Affiliations:** a Department of Cardiology, Heart Hospital, Hamad Medical Corporation, Doha, Qatar; b University of Michigan Health–Sparrow Heart and Vascular, and Michigan State University, Lansing, Michigan, USA

**Keywords:** intracardiac vegetation, Penumbra, right atrial mass, thrombectomy

## Abstract

**Background:**

Intracardiac masses traditionally require surgical resection, but transcatheter techniques offer a less invasive alternative.

**Case Summary:**

A 57-year-old woman with rheumatic heart disease and severe mitral stenosis was evaluated for percutaneous mitral commissurotomy. Imaging revealed a mobile right atrial mass near the inferior vena cava, posing a risk for embolization. Given the need for mass removal before the procedure, we performed the QATAR technique using the Penumbra Lightning 16 system, a loop snare, and electrocautery. The mass was successfully extracted without complications.

**Discussion:**

This case highlights the feasibility of a novel transcatheter approach for intracardiac mass removal, reducing the need for open surgery. The QATAR procedure integrates electrosurgery with catheter-based extraction, expanding therapeutic options for high-risk patients.

**Take-Home Messages:**

We aim to highlight a novel transcatheter technique combining electrosurgery and catheter-based therapies to remove intracardiac tumors. The QATAR procedure is a safe and effective technique for intracardiac mass removal, offering a minimally invasive alternative to surgery.

## Graphical Abstract

**Figure:**
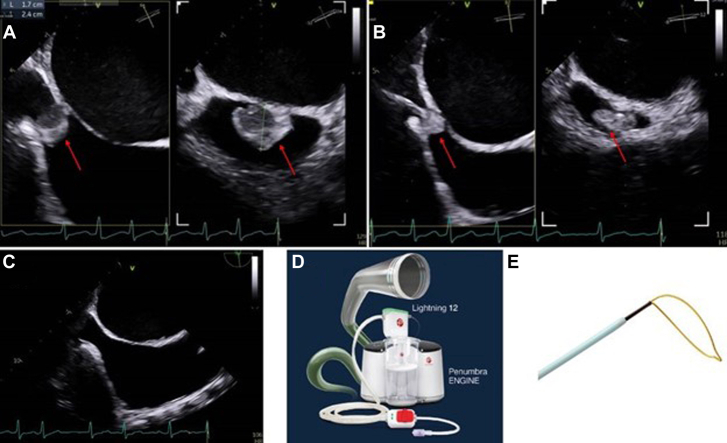

Intracardiac masses, which can be benign or malignant tumors, infections, or thrombi, are often seen attached to the heart’s inner lining.^1^ Traditionally, surgical removal has been the gold standard,^1^^,^^2^ but recent advances in transcatheter technology have made it possible to safely and effectively extract these masses, offering alternative options for patients.^1^, ^2^, ^3^, ^4^, ^5^

**Take-Home Messages**

• We aim to highlight a novel transcatheter technique combining electrosurgery and catheter-based therapies to remove intracardiac tumors.
• The QATAR procedure is a safe and effective technique for intracardiac mass removal, offering a minimally invasive alternative to surgery.

One such approach, The Simplified Extraction of Atrial Tumors with Targeted Loop Electricity (SEATTLE) procedure, has introduced a novel transcatheter method for removing small- to moderate-sized atrial tumors.^5^ However, when certain equipment is not available—in this case the ONO Endovascular Retrieval System (ŌNŌCOR LLC)—a modified version of this procedure—referred to as the QATAR technique—had to be improvised and used to extract a right atrial mass without resorting to surgery. This modification may serve as an alternative in select cases, and we hereby share our experience.

## History of Presentation

A 57-year-old African woman presented with worsening shortness of breath, fever, cough, and palpitations after an upper respiratory tract infection. On admission, the patient was tachycardic, tachypneic, hypoxic, and hypotensive, with bilateral crackles heard on auscultation.

## Past Medical History

Her history was notable for rheumatic heart disease with severe mitral stenosis, severe tricuspid regurgitation, and atrial fibrillation, all of which were managed medically for a while. Additionally, she had suffered an ischemic stroke and a subdural hematoma, requiring burr hole evacuation just 6 months before presentation. The patient has been maintaining anticoagulation with warfarin.

## Investigations and Management

Electrocardiogram showed atrial fibrillation with a rapid ventricular rate, and her laboratory work revealed elevated lactate levels (7 mmol/L). A respiratory viral panel confirmed influenza A infection, and she was initially treated with noninvasive ventilation, digoxin, diuretics, and antibiotics. Her condition was a clear exacerbation of decompensated heart failure secondary to severe mitral stenosis in addition to influenza A infection.

**Figure 1:**
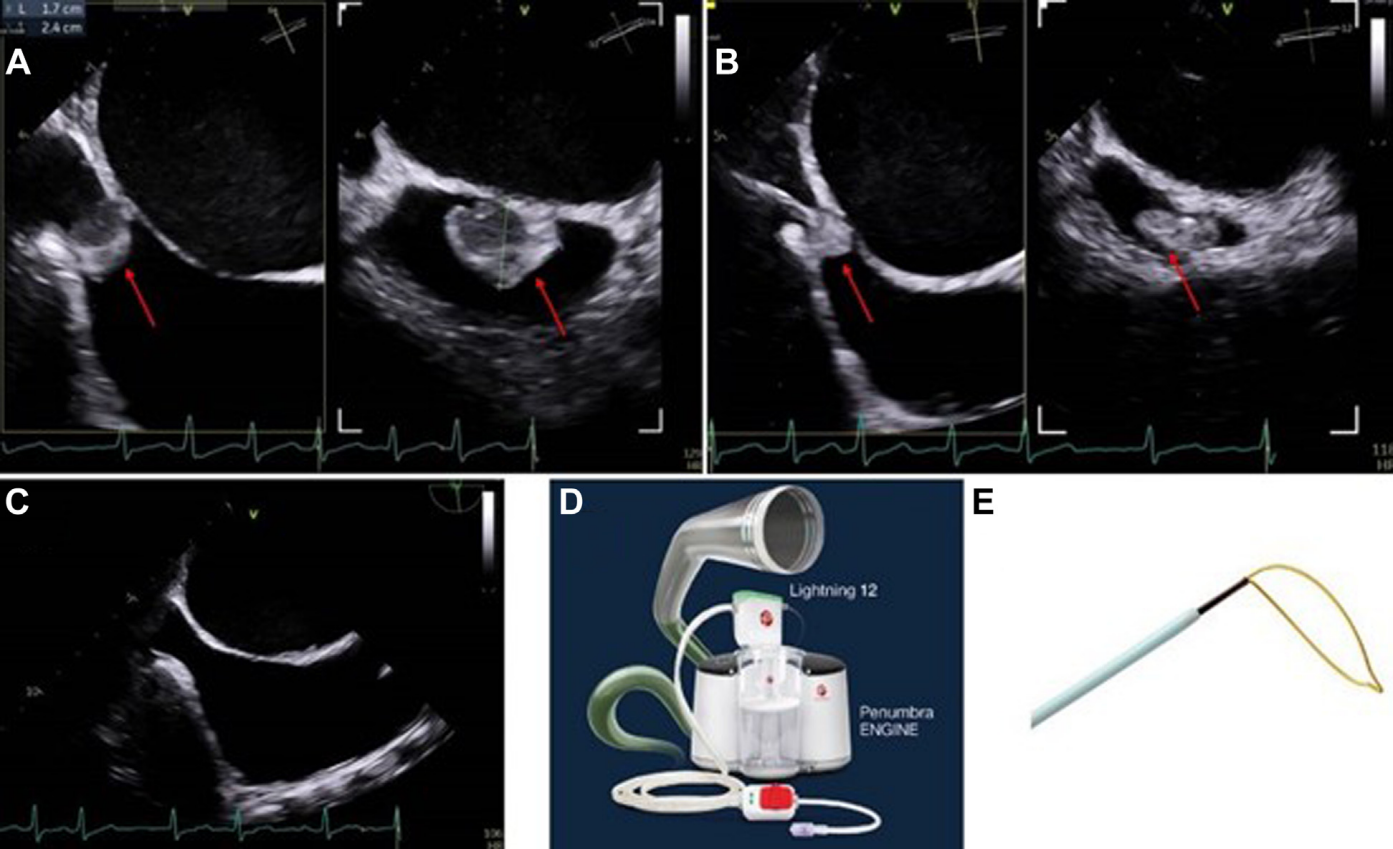
The QATAR Procedure (A) Transesophageal echocardiographic (TEE) bicaval view of a moderate-sized, pedunculated right atrial mass (red arrow) adherent to the interatrial septum. (B) TEE images of the Lightning Flash Indigo system, engaging the mass with negative pressure aspiration. (C) TEE images of right atrium after tumor extraction. (D) Lightning Flash Indigo system. (E) A loop snare device (Medtronic).

A transesophageal echocardiogram (TEE) revealed severe rheumatic mitral stenosis (mitral valve area: 0.8 cm^2^) with a Wilkins score of 9, mild mitral regurgitation, and severe tricuspid regurgitation. Additionally, TEE showed a mobile right atrial mass measuring 2.3 × 1.6 cm attached with a broad stalk near the inferior vena cava and coronary sinus (Figures 1A and 1B, Video 1). The differential diagnosis consisted of a tumor (eg, myxoma), an infectious vegetation, or a thrombus.

Because her condition stabilized from an infectious standpoint with negative blood cultures, she remained in heart failure and the heart team decided to proceed with percutaneous transvenous mitral commissurotomy. However, given the location of the mobile mass, it was decided that she needed the mass extracted beforehand.

## Mass Extraction Procedural Details

**Figure 2:**
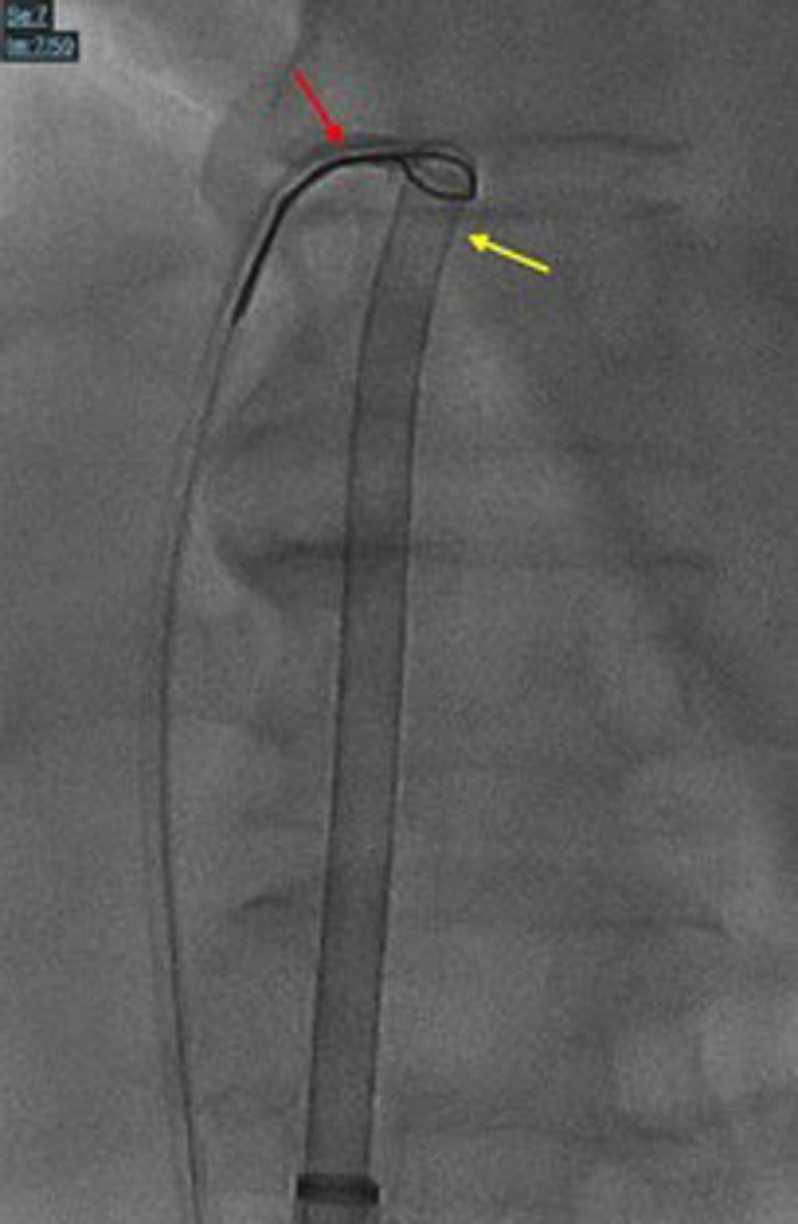
Fluoroscopy Image During the Procedure Fluoroscopy showing the snare (red arrow) in action encompassing the stalk of the mass along with Lightning Flash Indigo system (yellow arrow), engaging the mass with active suction.

**Figure 3:**
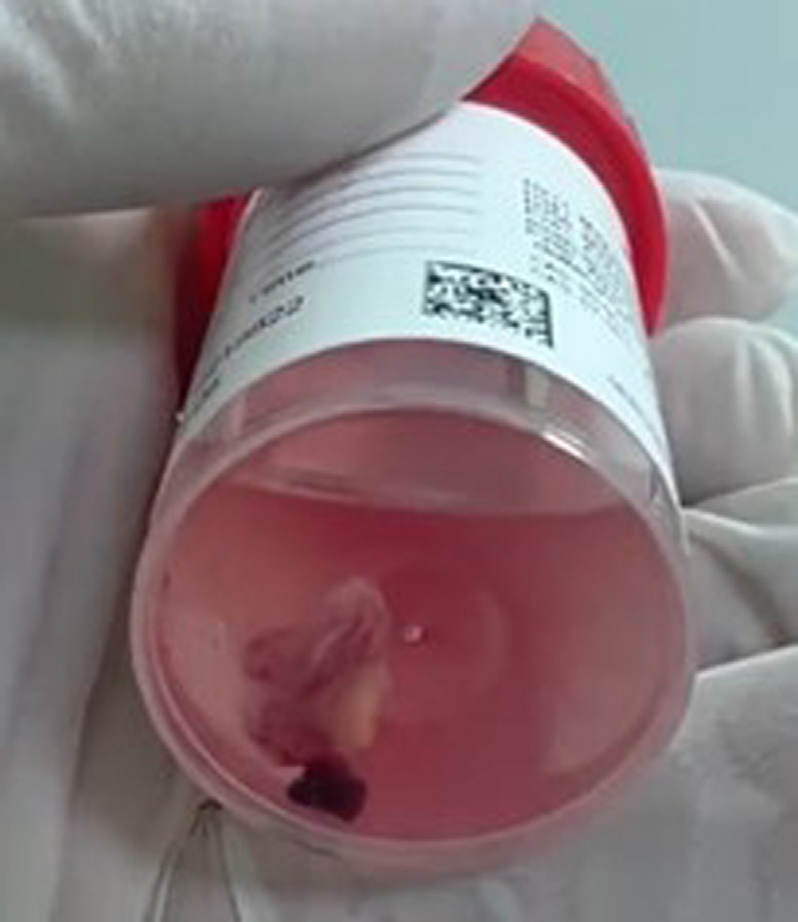
Macroscopic View of the Extracted Right Atrial Mass

Access was obtained in the right and left femoral veins and right radial artery. We used an 18-F Gore DrySeal sheath in the right vein and a 7-F standard sheath in the left vein. Due to the unavailability of the ONO Endovascular Retrieval System, a novel modification of the standard procedure was planned using the Penumbra Lightning Flash Indigo system (16-F). We first advanced a 10-mm Loop snare (Medtronic) inside a 6-F multipurpose guide into the inferior vena cava. Then, through the 16-F sheath, the Lightening 16 Penumbra was advanced inside of the snare and advanced into the right atrium. With fluoroscopy and live TEE guidance, the mass was engaged, and active suction was turned on (Videos 2 and 3). Due to the size and attachment of the mass, active aspiration from the Penumbra Lightening 16 was only enough to get a small piece out. Thus, the Loop snare was advanced over the Lightening 16 and over on the stalk, and the snare was tightened (Figure 2 and Videos 1 and 2 show the fluoroscopy/TEE loop of the actual cutting). A standard Bovie pencil was used to electrify the snare at 50-W pure cut while active aspiration was maintained. This allowed the stalk to be severed and the mass to be extracted en bloc safely without any complications (Figure 3, Video 4). The histopathology examination showed a benign simple cyst (blood cyst).

After the mass removal, attention shifted to the mitral valve, and percutaneous transvenous mitral commissurotomy was done successfully using standard techniques without any complications.

The patient tolerated the procedure well and was closely monitored and discharged without any complications.

## Outcome and Follow-Up

The patient successfully underwent the procedure without any postoperative complications. She was monitored in the hospital after the procedure. Echocardiography on the first postprocedural day showed a mean gradient of 4.2 mm Hg across the mitral valve and no residual intracardiac masses were seen.

## Discussion

Intracardiac masses are usually treated with surgical resection.^1^^,^^2^ However, several cases have recently demonstrated the successful use of transcatheter approaches for mass extraction for cardiac tumors.^3^, ^4^, ^5^, ^6^, ^7^, ^8^, ^9^

The QATAR procedure is an alternative simpler method of transcatheter mass extraction that can use Penumbra and a loop snare through 1 or 2 venous access sites. We think this method only works for smaller-sized masses; considering the use of 12- or 16-F Lightning catheters, we think that solid masses >2 cm should be reevaluated. Although a soft, fresh mass >2 cm may potentially be aspirated into the Penumbra, a more chronic, tough solid mass could become entangled at the top of the device.

Alternatively, the SEATTLE procedure uses more equipment: a steerable sheath, electrocautery snare and an En Snare, and an ONO retrieval basket (ŌNŌCOR LLC) for the endovascular removal of the mass (Table 1). However, we think that it can extract bigger-sized masses than the QATAR procedure technique.^5^

**Table 1:** QATAR and SEATTLE Comparison

Feature	QATAR	SEATTLE
Technical complexity	Simpler technically	More complex
Equipment and cost	Less equipment/potentially cheaper	More equipment needed/potentially more expensive
Lesion size	Good for small- and moderate-sized masses (up to 2 cm)	Good for all sizes (especially larger lesions)
Right-sided lesions	Works well; even with embolization, treatment can be done using the same device	Works well
Left-sided lesions	Usable with appropriate distal protection plans (simple setup)	Doable but more challenging due to complexity of setup; better embolization protection

SEATTLE = Simplified Extraction of Atrial Tumors with Targeted Loop Electricity.

Moreover, the theoretical risk of embolization in the QATAR procedure may be higher compared with the SEATTLE procedure due to the lack of protection from the ONO Endovascular Retrieval System. However, for smaller masses like the one in our case, the vacuum system effectively engulfed the mass into the Penumbra catheter, significantly reducing the risk.

Our group has published the MICHIGAN (Mass extraCtIon from the Heart FacIlitated by ONOCOR GlobAl embolic protectioN Device) procedure, using the ONO device in a unique manner through subclavian access and deploying the ONO in the ascending aorta acting as a whole-body embolic protection device.^4^ This approach helps mitigate potential embolization risks. However, the internal ONO catheter only accommodates a 7-F catheter (7-F Penumbra vs 7-F guide). This limitation may influence the feasibility of the approach in certain cases. Additionally, high-risk patients or those with certain mass features (eg, large, hardened, or calcified masses) may require further consideration before proceeding with extraction.

## Conclusions

The QATAR procedure provides a simple, innovative, and minimally invasive method for removing intracardiac masses, especially when specialized retrieval systems are unavailable. This case demonstrates the feasibility and safety of using this approach to extract intracardiac masses. We think this approach can also be used on the left side as well with careful planning and execution.

## Funding Support and Author Disclosures

The authors have reported that they have no relationships relevant to the contents of this paper to disclose.

## Appendix

Video 1 Transesophageal Echocardiogram: Bicaval View Before the Procedure

Video 2 During Mass Aspiration

Video 3 Loop Snare (Medtronic) Advanced Over the Lightening 16 and Over on the Stalk, and the Snare Was Tightened

Video 4 Transesophageal Echocardiogram: Bicaval View Post Mass Removal
